# Social context influences *Toxoplasma gondii* and *Trichinella* spp. infection in Alberta free-roaming wild pigs (*Sus scrofa*)

**DOI:** 10.1371/journal.pone.0324617

**Published:** 2025-05-23

**Authors:** Kiera Middel, Hailey Strydhorst, Hannah McKenzie, Chunu Mainali, Darcy R. Visscher

**Affiliations:** 1 Department of Biology, The King’s University, Edmonton, Alberta, Canada; 2 Alberta Agriculture and Irrigation, Edmonton, Alberta, Canada; 3 Department of Biological Science, University of Alberta, Edmonton, Alberta, Canada; Guru Angad Dev Veterinary and Animal Sciences University, INDIA

## Abstract

The increasing spread of wild pigs across Canada is concerning due to their potential role as reservoirs for zoonotic diseases, including trichinosis and toxoplasmosis. Trichinosis is caused by the parasitic nematode *Trichinella* spp. and can manifest clinically in humans. Likewise, the intracellular protozoan parasite *Toxoplasma gondii* is a significant global foodborne and waterborne parasite responsible for toxoplasmosis. Despite wild pigs being recognized globally as reservoirs for *T. gondii* and *Trichinella* spp., the status of wild pigs in Alberta remains undetermined. Wild pig diaphragms were collected as part of provincial control efforts between 2018–2024 from four Alberta counties. Meat juice was analyzed for antibodies against *Trichinella* spp. and *T. gondii* using commercial ELISA kits. We found that 27/252 pigs were positive for antibodies against *T. gondii* and 11/293 positive for antibodies against *Trichinella* spp., corresponding with the respective true prevalence of 15.3% (95% CrI: 9.6–22.7%) and 3.8% (95% CrI: 1.8–6.5%) based on Bayesian analysis using reported test sensitivity and specificity. We found a difference in true prevalence of antibodies against *Trichinella* spp. between counties, and the prevalence of antibodies against *Trichinella* spp. and *T. gondii* was significantly higher in solitary wild pigs, suggesting social context of the individual is an important determinant of infection risk. These findings suggest that Alberta wild pigs function as *Trichinella spp.* and *T. gondii* reservoirs, underscoring the need for a One Health approach for managing Alberta’s invasive wild pigs.

## Introduction

Wild pigs (*Sus scrofa*)—known by various names such as wild boar, wild/feral swine, wild/feral hog, and feral pig—are highly invasive. Despite the phenotypic variation within the *Sus scrofa* species, universal features of wild pigs include their adaptable biology, generalist diet, and rapid reproduction rate [1,2]. These characteristics render wild pigs a formidable invasive species, as evidenced by their near-global distribution and their increasing spread documented in various countries, including Canada [1,3,4]. Wild pigs were initially introduced to Canada in the 1980s as part of livestock diversification initiatives and have since spread, particularly in the western prairie provinces, including Alberta, through escapes and releases from farms [3,5,6]. Their increased presence is concerning due to their negative ecological impacts as well as their ability to carry and transmit many diseases of concern for wildlife, livestock, and humans, including hepatitis E, tuberculosis, *Echinococcus multilocularis,* African swine fever virus, trichinosis, and toxoplasmosis [7–9].

Trichinosis or trichinellosis is a disease caused by the parasitic nematode *Trichinella* spp. through consumption of viable larvae in infected meat [10,11]. The genus is composed of 13 species with 10 occurring as encapsulated species and 3 as non-encapsulated species, each species having different geographical ranges and host preferences [12,13]. Globally and historically, domestic swine are primary sources of human trichinosis [14]. However, biosecurity practices have eliminated infection in Canadian commercial domestic swine [14–17]. Wild pigs are known as prominent sources of trichinosis due to their opportunistic scavenging diet exposing them to sylvatic *Trichinella* spp. via tissue cysts in infected carcasses [18,19]. Human outbreaks in Canada, however, have only been linked to backyard-raised pigs or, more frequently, the consumption of bear meat [18–26].

Toxoplasmosis is a disease caused by the intracellular protozoan parasite *Toxoplasma gondii,* which utilizes warm-blooded animals as intermediate hosts and the family Felidae for definite hosts [27,28]. *T. gondii* is one of the leading foodborne pathogens causing hospitalization and death [29,30]. While most infections remain asymptomatic, *T. gondii* poses a significant risk to public health, particularly for immunocompromised individuals and pregnant women [31,32]. Latent infections can still have economic and public health implications, as studies have demonstrated correlations between infection status and mental health conditions, entrepreneurship, and traffic accidents as a result of increased risk taking behaviour [33–38].

Humans and other intermediate hosts can contract *T. gondii* through various pathways, though undercooked meat remains a dominant source of human toxoplasmosis [27,39]. While domestic livestock still exhibit low seroprevalence against *T. gondii*, wild game meat is a more common contemporary source of human disease, particularly wild pig and deer meat [40–48]. Given their omnivorous diet, which incorporates rooting and scavenging, wild pigs are exposed to multiple routes of infection, making them an ideal bioindicator species for sylvatic *T. gondii* [49,50].

Despite the increasing spread of wild pigs in Canada and their recognized role elsewhere as reservoirs of *Trichinella* spp*.* and *T. gondii*, the prevalence of these parasites in Alberta wild pig populations remains poorly understood. Additionally, the impact of wild pig social context on the prevalence of these parasites remains elusive, as most studies have focused on individual hunted wild pigs. Given this knowledge gap, our study aims to assess the prevalence and associated risk factors, including social context, for the presence of antibodies against *Trichinella* spp*.* and *T. gondii* in Alberta’s wild pig population.

## Methods

### Sample collection

Samples were collected from wild pigs removed through provincial control efforts in Alberta. Whole sounder trapping was used to capture entire sounders, or groups of wild pigs, in large corral traps at locations identified to contain wild pigs based on habitat disturbances and/or complaints about agricultural damage. Corral doors were remotely closed on sounders by operators who had live images of the trap to maximize the chance that an entire sounder was captured. In a few instances only individual wild pigs were captured after setting up the corral trap and sounders were not present. The captured animals were euthanized and sent to the provincial Animal Health and Assurance Branch Postmortem Facility, where tissue samples and additional descriptive information was collected, including morphological measurements, sex, and age. For this study a total of 293 wild pig diaphragms, commonly used for parasitological research and for the extraction of meat juice, were collected from 10 sites across four counties (Strathcona County, Two Hills County, Woodlands County, and Lac Ste Anne County). A total of 279 wild pigs were captured in sounder groups (≥2 wild pigs) and 14 were captured as solitary wild pigs. The King’s University Animal Care committee determined that this study, using stored samples, did not require explicit approval.

### ELISA testing

Meat juice was collected from the diaphragms and tested in duplicate for the presence of antibodies to *Trichinella* spp. and *T. gondii* using two commercially available enzyme-linked immunosorbent assay (ELISA) kits (ID Screen® Toxoplasmosis Indirect Multi-species (IDvet, Grabels, France); PrioCHECK® Trichinella Ab, Applied Biosystems, ThermoFisher Scientific, Lelystad, The Netherlands). The tests were conducted following the manufacturers’ protocol for meat juice samples. Results were calculated as a mean of the duplicates and positives were required to have a coefficient of variation (CV) below 20%.

For the *T. gondii* tests, the sample-to-positive control percentage (SP%) was calculated based on the manufacturer’s equation:


$$\begin{array}{c} \text{SP}\%\;=\frac{OD_{sample}-OD_{Negative\;Control}}{OD_{Postive\;Control}-OD_{Negative\;Control}}\;x100 \end{array}$$ (1)

Samples with SP% ≤ 40% were considered negative, between 40% and 50% as doubtful, and ≥ 50% as positive, as suggested by the manufacturer.

Similarly, with the *Trichinella* spp. ELISA tests, the percent positivity (PP) was calculated using the provided equation:


$$PP=\frac{OD_{450\;Sample}}{OD_{450\;Positive\;Control}}x100$$ (2)

Samples with a PP value equal to or over 15% were classified as positive cases while those with PP values below 15% were considered negative cases, as suggested by the manufacturer.

The diaphragm samples (3.5g-37g) that were ELISA positive or partial positive (only one duplicate was positive) for *Trichinella* spp. were sent for additional testing at the Canadian Food Inspection Agency’s Centre for Food-borne and Animal Parasitology (Saskatoon, Canada). A pepsin/hydrochloric acid artificial digestion was utilized for isolating larva [51]. A larva morphologically consistent with *Trichinella* spp. was isolated from one sample but was too degraded for PCR.

### Data analysis

All statistical analysis was performed using R Studio Software (version 4.4.1). To address the imperfect nature of the ELISA, the true prevalence was estimated using a Bayesian approach with the ‘prevalence’ package in R [52,53]. The sensitivity and specificity were derived from the reported kit values and incorporated as uniform prior distributions. The PrioCHECK® Trichinella Ab kit has the reported respective sensitivity and specificity of 97.1%-97.8% and 99.5%-99.8%, while the ID Screen® toxoplasmosis kit has the respective sensitivity and specificity of 57.3%-87% and 99.4%-100% [54–57]. In this model, two chains of 20,000 iterations were stimulated using the sensitivity distribution, of which the first 10,000 were discarded as “burn-in” samples [58]. Convergence of the model was ensured using the Brook-Gelman-Rubin statistic, and the model yielded the 95% credible intervals (CrI) estimates.

We conducted Pearson’s Chi-squared test to examine prevalence differences among counties, age groups, sexes, date of sampling (year), and social context. The wild pigs were classified as solitary if they were captured as individuals, and as part of a sounder if they were captured in a group of two or more wild pigs. Only one sample was collected from 2018, and thus was removed for the sampling date analysis. One sample was collected from Elk Island National Park (EINP), but for county analysis it was categorized as being from Strathcona County, the county within which the park is embedded. The results were visualized and mapped in R.

## Results

A total of 252 wild pig samples yielded sufficient meat juice and were tested for antibodies against *T. gondii*, of which 27 were positive, corresponding with an apparent prevalence of 10.71% (95% CrI: 7.18–15.2%) and a calculated true prevalence of 15.3% (95% CrI: 9.6–22.8%). Following individual-level analysis, there was no significant difference in *T. gondii* prevalence according to age, sex, sampling date or county (Table 1). The solitary wild pig collected from EINP was positive for antibodies against *T. gondii*.

**Table 1:** Univariate analysis of associated risk factors for presence of antibodies against *T. gondii.*

	Number Positive	True Prevalence (95% CrI)	X^2^	Degrees of freedom	P-value
**Sex**					
Female	13/133	14.4% (7.4-24.0%)	0.09	1	0.76
Male	14/119	17.3% (9.2-27.7%)			
**Age**					
Mature	16/177	13.2% (7.3-20.8%)	1.21	1	0.27
Juvenile	11/75	21.9% (11.1-36.5%)			
**County**					
Strathcona	1/9	25.7% (3.1-65.4%)	6.68	3	0.08
Lac Ste Anne	3/50	10.6% (2.6-23.3%)			
Woodlands	18/175	14.9% (8.5-23.3%)			
Two Hills	5/18	42.8% (16.9-76.4%)			
**Date**					
2021	10/85	17.7% (8.6-30.0%)	6.54	3	0.09
2022	5/69	11.8% (4.0-23.2%)			
2023	6/24	38.1% (16.6-66.7%)			
2024	6/73	13.0% (4.9-24.6)			
**Social Context**					
Sounder	12/164	10.8% (5.4-18.3%)	5.46	1	0.02
Solitary	4/13	47.0% (17.3-85.0%)			

Similarly, 11 out of 293 wild pigs tested positive for antibodies against *Trichinella* spp., resulting in an apparent prevalence of 3.75% (95% CrI: 1.89–6.62%) and a calculated true prevalence of 3.8% (95% CrI:1.8–6.5%). There was no significant difference in *Trichinella* spp. prevalence according to age, sex, or sampling date, but a significant difference was found for county (Table 2).

**Table 2:** Univariate analysis of associated risk factors for presence of antibodies against *Trichinella* spp.

	Number Positive	True Prevalence (95% CrI)	X^2^	Degrees of freedom	P-value
**Sex**					
Female	7/157	4.8% (1.9-8.7%)	0.14	1	0.71
Male	4/132	3.5% (0.9-7.5%)			
**Age**					
Mature	7/198	3.7% (1.5-6.9%)	<0.01	1	1
Juvenile	4/95	4.9% (1.4-10.3%)			
**County**					
Strathcona	0/11	7.8% (0.00-22.5%)	8.53	3	0.04
Lac Ste Anne	3/52	7.3% (1.8-15.8%)			
Woodlands	5/209	1.2% (0.1-3.6%)			
Two Hills	3/21	17.6% (4.9-35.7%)			
**Date**					
2021	3/100	3.7% (0.8-8.5%)	2.05	3	0.56
2022	2/83	3.3% (0.4-8.1%)			
2023	2/25	11.2% (2.2-25.6%)			
2024	4/84	5.6% (1.6-11.7%)			
**Social Context**					
Sounder	4/183	2.4% (0.60-5.3%)	8.21	1	<0.01
Solitary	3/15	24.0% (7.3-46.8%)			

**Fig 1:**
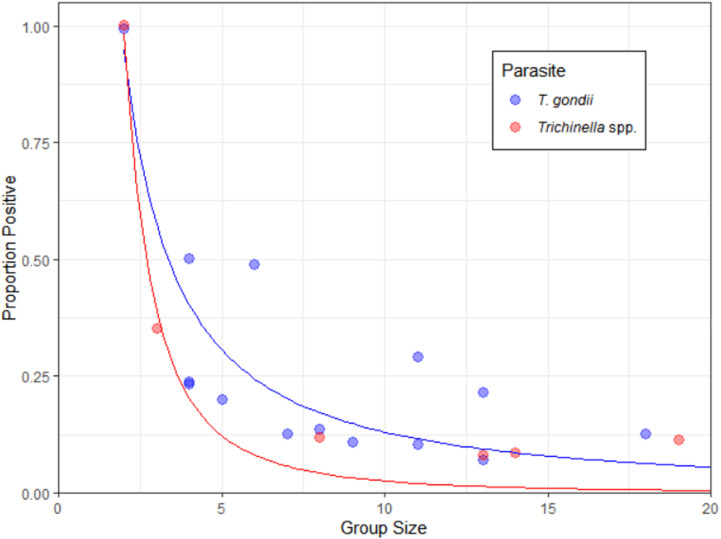
Proportion positive in sounders with 1 or more wild pigs positive for antibodies against *Trichinella* spp. or *T. gondii.* The blue line indicates the trendline for *T. gondii* following the equation y = 2.226x^-1.233^ (R^2^ = 0.76), while the red line follows the trendline for *Trichinella* spp. with the equation, y = 4.745x^-2.272^ (R^2^ = 0.96).

A total of 36 sounders were analyzed with 6 sounders having up to two wild pigs positive for antibodies against *Trichinella* spp., and 14 sounders having 1–3 wild pigs positive for *T. gondii*. For both parasites, the sounders containing positive wild pigs demonstrated increased prevalences in smaller sounder groups, with infection often occurring in clumps (Fig 1).

**Fig 2:**
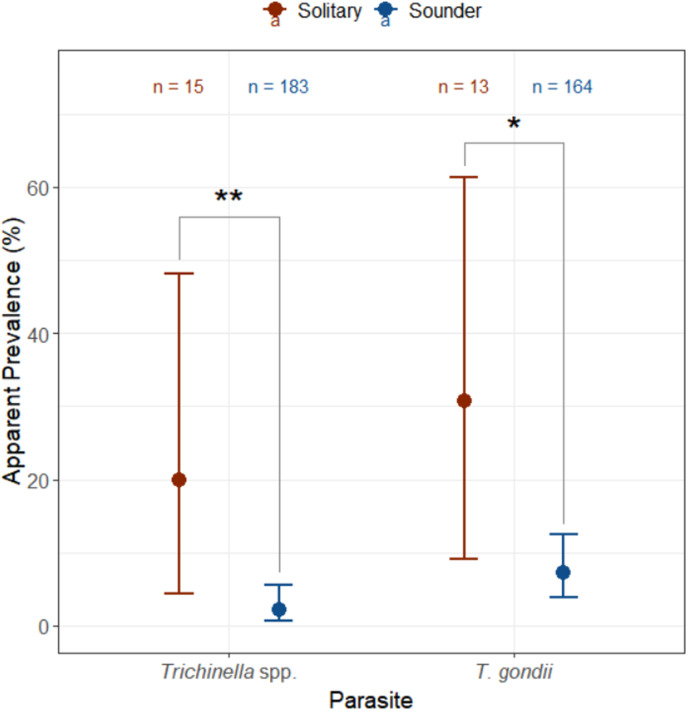
Apparent prevalence of antibodies against *Trichinella* spp. and *T. gondii* in mature solitary and sounder wild pigs. A statistically significant difference in prevalence between social context was found for both *Trichinella* spp. (X^2^ = 9.06, df = 1, p-value < 0.01) and *T. gondii* (X^2^ = 5.46, df = 1, p-value = 0.02). The ‘*’ indicates a p-value < 0.05, and ‘**’ indicates p-value < 0.01.

Analysis found that mature solitary wild pigs had significantly higher prevalence compared to mature sounder wild pigs in both *Trichinella* spp. (X^2 ^= 9.06, df = 1, p-value < 0.01) and *T. gondii* (X^2^ = 5.46, df = 1, p-value = 0.02), as demonstrated in Fig 2.

Though three wild pigs were infected with both parasites, there was no association between *Trichinella* spp. and *T. gondii* infection status (X^2^ = 1.73, df = 1, p-value = 0.19). The three wild pigs with co-infection included a small sounder group (n = 2) where both pigs were positive for antibodies against *Trichinella* spp. and *T. gondii.*

## Discussion

This study provides serological evidence of *T. gondii* and *Trichinella* spp. infection in Alberta wild pigs. Our prevalences are lower than the global seroprevalence in wild pigs, *T. gondii* infection is estimated at 23% while *Trichinella* spp. infection is approximated at 6% [19,48]. However, prior to this research, there was no indication of either *T. gondii* or *Trichinella* spp. infection in free ranging Canadian wild pigs. The only previous research is a 2014 Saskatchewan study, which found no evidence of *T. gondii* or *Trichinella* spp. in their small sample (n = 22) of wild pigs using PCR techniques [59]. Our results indicate that the prevalence of *Trichinella* spp. significantly varied among counties in Alberta, while there was a nonsignificant trend for a difference in *T. gondii* prevalence (Tables 1 and 2). There is an interesting east-west trend where the eastern-most county has the highest prevalence of both *T. gondii* and *Trichinella* spp., but this may be a result of relatively few counties in Alberta being sampled. However, counties were sampled based on persistent reports of wild pig presence and agricultural damage and while constituted relatively few counties, likely encompass the core range of wild pigs in Alberta. Recent research from the US indicates an increase in seroprevalence of both anti-*Trichinella* and anti-*T. gondii* antibodies in wild pigs, found to be 12.4% and 40.8% respectively, and suggested that the rise in seroprevalence for both parasites may be linked to expanding populations of susceptible wild pigs [60]. If this trend holds, and our results are generalizable, then there ought to be concern for the continued spread of wild pigs in Canada along with the zoonotic parasites they may carry.

The artificial digestion isolated only one larva morphologically consistent with *Trichinella* spp., but unfortunately the species could not be determined. One potential reason for the low evidence of larvae could be false positives due to poor specificity of the ELISA test for wild pigs or that most diaphragm samples had a low mass (below 5g) which made detecting a worm burden difficult [55,61]. Alternatively, the wild pigs may have been infected with a *Trichinella* spp. incompatible with wild pig hosts, in which the larvae failed to persist in the muscle but still induced a detectable antibody response, indicating exposure without subsequent infection.

Canada is known to host a variety of *Trichinella* spp. infected wildlife, although the *Trichinella* spp. most associated with the wild pig hosts, *T. spiralis* and *T. pseudospiralis*, have rarely been identified in Canada’s sylvatic cycle [12,62–66]. Conversely, the *Trichinella* spp. frequently found in Canada, *T. nativa* and T6, are largely incompatible with wild pigs hosts and have few reports of natural infection [63,65,67,68]. In experimental studies, both *T. nativa* and T6 demonstrated lower larva burden, decreased persistence of larva cysts in muscle, and reduced detectable antibody response [63,68–70]. In addition, while the duration of anti-*Trichinella* spp. antibodies during infection remains variable, it is agreed that they can persists in samples with no recoverable muscle larvae [69–71]. Therefore, it is possible that at least some of the ELISA positives in this study were true positives detecting the antibodies from a past *Trichinella* spp. infection.

Due to the capture of whole sounders and solitary wild pigs, our study had the unique opportunity to analyze toxoplasmosis and trichinosis infection within different social groupings. We sampled only 15 total solitary wild pigs, four of which were female [72]. This was, in part, a bias of the corral trapping locations being situated where wild pigs had been reported to cause damage, something more likely to occur when a sounder is present compared to a solitary individual. While we can’t definitively identify individuals as not being part of a sounder their capture came not finding a sounder in the area and individuals were not captured in the same site as recent sounder captures (i.e., they were not individuals “missed” in the initial sounder capture). Nonetheless, we found that solitary wild pigs had significantly higher prevalence of antibodies against *T. gondii* and *Trichinella* spp., identifying them as potential sylvatic reservoirs for both parasites (Fig 2). In general, solitary wild pigs exhibit elevated movement, though this effect has primarily been studied only in males, presumably increasing potential exposure to the parasites [72,73]. However, *T. gondii* has also demonstrated the ability to induce behavioural changes in various mammals, including risk-taking [74,75]. While the simultaneous elevation of *Trichinella* spp. and *T. gondii* infection suggests that solitary behaviour heightens exposure to these parasites, this does not rule out the possibility that toxoplasmosis could enhance the preference for the riskier solitary lifestyle [74,75]. Our results highlight solitary wild pigs as important sylvatic reservoirs for these parasites, and indicates their potential as disease vectors, particularly for any dispersing solitary wild pigs. Solitary wild pigs, as potential disease vectors, warrant further study and may require different management strategies compared to whole sounder captures if they become the focus of disease control efforts due to their relatively high prevalence of *Trichinella* spp. and *T. gondii* and potential as a long-distance transmitter of the parasites.

Our analysis of infection within wild pig sounders revealed that infection was not homogenous: only a portion of the groups contained infected individuals, and these infections occurred in clusters of 1–3 wild pigs (Fig 1). While various studies have explored the relationship between social context and disease prevalence for non-trophic transmissible diseases, there is limited research on trophic-transmissible diseases [76–78]. Since *T. gondii* and *Trichinella* spp. are not contact transmissible, instead requiring predation or necrophagy, it is not surprising that only a few individuals within each sounder group were infected. However, the clustering of infections suggests that social context may influence infection, with proximity leading to shared resources either a small carcass or, alternatively, social dominance behaviour within the sounder resulting in differential exposure at a contaminated source [79,80]. These findings highlight that social organization may play a role in infection dynamics, potentially facilitated by unequal sharing of contaminated resources.

An interesting case was a positive solitary male from EINP, a fully fenced national park embedded within the county of Strathcona containing various ungulates [81]. Ungulates are common sylvatic carriers of *T. gondii*, and, European bison (*Bison bonasus bonasus*) are known to spontaneously abort due to *T. gondii* infection [82,83]. Whether the wild pig acquired *T. gondii* elsewhere and represents a transmission risk to EINP or became infected within EINP, both possibilities imply potential for sylvatic *T. gondii* within the park and could have negative repercussions for disease management and the protection of ungulates in EINP.

Due to their opportunistic omnivore diet, involving scavenging and rooting behaviour, wild pigs experience exposure to *T. gondii* through both environmental oocysts and tissue cysts [50,84]. These characteristics make them valuable as an indicator species for sylvatic *T. gondii*, reflecting environmental oocyst contamination and infection among spatially co-occurring wildlife in Alberta. Current research on the *T. gondii* status of Alberta wildlife is limited and outdated, with few species being found to have infections [85–88]. Our finding of *T. gondii* in Alberta free-ranging wild pigs reveals a current presence of sylvatic *T. gondii* in Alberta and suggest future studies are warranted.

While the evidence of *Trichinella* spp. in Alberta wild pigs does indicate the presence of sylvatic *Trichinella* spp. in Alberta wildlife, wild pigs as indicator species may be under-representing the parasite’s presence. Firstly, as opportunistic scavengers, wild pigs are not exposed to tissues cysts to the same extent as carnivorous sentinels [64]. Furthermore, the *Trichinella* spp. documented in Canada are largely incompatible with wild pig hosts, and thus Alberta wild pigs may not indicate *Trichinella* spp. presence following exposure to viable larvae [63,68–70]. Nevertheless, the anti-*Trichinella* spp. antibodies in Alberta wild pigs demonstrates the current existence of sylvatic *Trichinella* spp. in spatially co-occurring wildlife, which is highlighted by the positive seroprevalence observed in various Alberta species [62,65,89].

The presence of *T. gondii* and *Trichinella spp*. in Alberta wild pigs raises concerns about potential risks for consumers of wild pig meat. Indeed, human cases of both trichinosis and toxoplasmosis have been associated with wild pigs [10,18,40]. The presence of sylvatic *Trichinella* spp. and *T. gondii* raises further concerns regarding other Alberta wildlife species. Multiple toxoplasmosis outbreaks have been associated with venison worldwide, and bear meat is a prominent source of human trichinosis in Canada [20–22,24–26,42,44–47]. In addition, one study revealed the potential for *T. gondii* infection in non-consumers following direct contact with infected blood, demonstrating the importance of following food-safe procedures when dressing and processing wild game meat, particularly wild pigs [46].

The trophic transmission required for wild pigs to directly infect another animal, with either parasite, suggests that direct infection to livestock is improbable, however, wild pigs could act as sources of infection for synanthropic species [90]. *T. gondii* is not uncommon in livestock, and while extremely rare, *Trichinella* spp. has also been documented in domestic animals [18,39,43,91]. As a result, care should be taken when consuming non-biosecure animals, especially in locations known to be occupied by free-roaming wild pigs who may pose a transmission risk.

## Conclusion

This study found evidence of both *T. gondii* and *Trichinella* spp. infection in Alberta wild pigs and illuminates the current sylvatic presence of these parasites in the Alberta environment. Solitary wild pigs were more likely to be infected and serve as reservoirs for these parasites, which implicates their importance for consideration of disease spread and control. This new finding underscores the importance of adopting a collaborative One Health Approach for managing toxoplasmosis and trichinosis, particularly regarding the implicated risk of invasive wild pigs. However, the study only occurred across a narrow geographical range within Alberta, so further research across Canada is needed to fully understand the scope of *T. gondii* and *Trichinella* spp*.* infection status in wild pigs throughout Canada. Future studies specific to *T. gondii* could consider the potential behavioural changes of toxoplasmosis and the resulting role of *T. gondii* in the wild ecosystem processes.

## Supporting information

S1 Table Data file of for wild pigs included in the analysis. Table of data for individual wild pigs included in the analysis including their infection status and demographic parameters.
(PDF)
