# Supplementary material for: Social context influences Toxoplasma gondii and Trichinella spp. infection in Alberta free-roaming wild pigs (Sus scrofa)
Source: PLoS One. 2025 May 23;20(5):e0324617. doi: 10.1371/journal.pone.0324617 (PMC12101635; doi:10.1371/journal.pone.0324617)
Supplement: S1 Table — Table of data for individual wild pigs included in the analysis including their infection status and demographic parameters. (PDF) [file pone.0324617.s001.pdf]

ID	groupsize	county	sex	age	weight	length	date	sounder	toxo	trich
1	3	Woodlands	M	M	62	97	2018	S	0	0
2	14	Woodlands	F	M	34	103	2021	S	0	0
3	14	Woodlands	M	M	30	103	2021	S	0	0
4	14	Woodlands	F	M	26	104	2021	S	0	0
5	14	Woodlands	M	M	49	123	2021	S		0
6	14	Woodlands	F	M	30	99	2021	S		0
7	14	Woodlands	M	M	27	91	2021	S	0	1
8	14	Woodlands	F	M	27	97	2021	S		0
9	14	Woodlands	F	M	31.5	102	2021	S	0	0
10	14	Woodlands	F	M	28	99	2021	S	0	0
11	14	Woodlands	M	M	25	96	2021	S	0	0
12	14	Woodlands	F	M	30.5	100	2021	S	0	0
13	14	Woodlands	F	M	26	98	2021	S	0	0
14	14	Woodlands	F	M	26	96.5	2021	S	1	0
15	14	Woodlands	F	M	27.5	98	2021	S	0	0
16	5	Woodlands	M	M	26	80	2021	S	0	0
17	5	Woodlands	F	M	22	92	2021	S	0	0
18	5	Woodlands	F	M	27	95	2021	S		0
19	5	Woodlands	M	M	26	101	2021	S	0	0
20	5	Woodlands	M	M	26	105.5	2021	S	0	0
21	7	Lac Ste Anne	F	M	110	152	2021	S	0	0
22	7	Lac Ste Anne	M	M	53	120	2021	S	0	0
23	7	Lac Ste Anne	F	M	27	104	2021	S	0	0
24	7	Lac Ste Anne	F	M	44	119	2021	S	0	0
25	7	Lac Ste Anne	F	M	46	115	2021	S	0	0
26	7	Lac Ste Anne	F	M	48	119.5	2021	S	0	0
27	7	Lac Ste Anne	M	M	46	121	2021	S	0	0
28	11	Woodlands	F	M	44	116	2021	S	0	0
29	11	Woodlands	F	M	27	99	2021	S	0	0
30	11	Woodlands	F	M	28	96.5	2021	S	0	0
31	11	Woodlands	F	M	25.5	98.5	2021	S	0	0
32	11	Woodlands	F	M	28.5	101	2021	S	0	0
33	11	Woodlands	M	M	28.5	97	2021	S	0	0
34	11	Woodlands	M	M	25	93.5	2021	S	0	0
35	11	Woodlands	F	M	43	113	2021	S	0	0
36	11	Woodlands	M	M	45.5	116.5	2021	S	0	0
37	11	Woodlands	M	M	25.5	95	2021	S	0	0
38	11	Woodlands	M	M	44	109.5	2021	S	0	0
39	19	Lac Ste Anne	F	M	86	152	2021	S		0
40	19	Lac Ste Anne	F	M	83	150	2021	S	0	0
41	19	Lac Ste Anne	F	J	6	59	2021	S	0	0
42	19	Lac Ste Anne	M	J	10	71	2021	S	0	0
43	19	Lac Ste Anne	M	J	6	61	2021	S	1	0
44	19	Lac Ste Anne	F	J	5	58	2021	S	1	0
45	19	Lac Ste Anne	F	J	5	58	2021	S	0	1
46	19	Lac Ste Anne	M	J	8	61	2021	S	0	0
47	19	Lac Ste Anne	F	J	7	58	2021	S	0	0
48	19	Lac Ste Anne	M	J	10	68	2021	S	0	0
49	19	Lac Ste Anne	F	J	9	69	2021	S	0	0
50	19	Lac Ste Anne	M	J	8	61	2021	S	0	0
51	19	Lac Ste Anne	F	J	5	62	2021	S	0	0
52	19	Lac Ste Anne	M	J	7	64	2021	S	0	0
53	19	Lac Ste Anne	F	J	6.5	64	2021	S	0	0
54	19	Lac Ste Anne	M	J	8	63	2021	S	0	0
55	19	Lac Ste Anne	M	J	6	59	2021	S	0	1
56	19	Lac Ste Anne	F	J	5	57	2021	S	0	0
57	19	Lac Ste Anne	M	J	7	65	2021	S	0	0
58	14	Woodlands	M	M	26	95	2021	S	0	0
59	14	Woodlands	F	M	38	100	2021	S	0	0
60	14	Woodlands	F	J	4	52	2021	S	0	0
61	14	Woodlands	F	J	6	55	2021	S	0	0
62	14	Woodlands	F	J	5	53	2021	S	0	0
63	14	Woodlands	F	J	8	65.5	2021	S	0	0
64	14	Woodlands	M	J	9	67	2021	S	0	0
65	14	Woodlands	M	J	6	56	2021	S		0
66	14	Woodlands	F	J	8	65	2021	S		0
67	14	Woodlands	F	J	6	59	2021	S	1	0
68	14	Woodlands	M	J	6	57	2021	S	1	0

69	14	Woodlands	M	J	6	59	2021	S	0	0
70	14	Woodlands	M	J	6	60	2021	S	1	0
71	14	Woodlands	F	J	6	59	2021	S		0
72	20	Woodlands	M	M	41	116	2021	S	0	0
73	20	Woodlands	F	M	85	145	2021	S		0
74	20	Woodlands	F	M	83	146	2021	S		0
75	20	Woodlands	F	M	83	144	2021	S	1	0
76	20	Woodlands	F	J	7	62	2021	S		0
77	20	Woodlands	F	J	6	54	2021	S		0
78	20	Woodlands	F	J	12	69	2021	S	0	0
79	20	Woodlands	M	J	8	63	2021	S	0	0
80	20	Woodlands	F	J	7	56	2021	S	0	0
81	20	Woodlands	F	J	7	59	2021	S		0
82	20	Woodlands	F	J	11	73	2021	S	0	0
83	20	Woodlands	F	J	10.5	71	2021	S	0	0
84	20	Woodlands	F	J	10	67	2021	S	0	0
85	20	Woodlands	F	J	7.5	60	2021	S	0	0
86	20	Woodlands	M	J	5	53	2021	S		0
87	20	Woodlands	M	J	11.5	73	2021	S	0	0
88	20	Woodlands	M	J	12.5	75	2021	S	0	0
89	20	Woodlands	M	J	8.5	63	2021	S	1	0
90	20	Woodlands	M	J	6	56	2021	S		0
91	20	Woodlands	M	J	5	51	2021	S	1	0
92	4	Woodlands	F	M	40		2021	S	0	0
93	4	Woodlands	M	M	34		2021	S	0	0
94	4	Woodlands	M	M	39		2021	S	0	0
95	4	Woodlands	M	M	44		2021	S	0	0
96	1	Woodlands	M	M	67	130	2021	I	0	0
97	5	Woodlands	F	M	102	149	2021	S	1	0
98	5	Woodlands	F	M	93	156	2021	S	0	0
99	5	Woodlands	M	M	41	111.5	2021	S	0	0
100	5	Woodlands	F	M	92	138	2021	S	0	0
101	5	Woodlands	F	M	97	136	2021	S	0	0
102	13	Lac Ste Anne	F	M	107	161.5	2022	S	0	0
103	13	Lac Ste Anne	F	M	107	159.5	2022	S	0	0
104	13	Lac Ste Anne	F	M	75	141	2022	S	0	0
105	13	Lac Ste Anne	F	M	68	136	2022	S	0	0
106	13	Lac Ste Anne	F	M	40	111.5	2022	S	0	0
107	13	Lac Ste Anne	F	M	45	118	2022	S	0	1
108	13	Lac Ste Anne	M	M	42	110	2022	S	0	0
109	13	Lac Ste Anne	M	M	44	120.5	2022	S	0	0
110	13	Lac Ste Anne	M	M	47	107	2022	S	0	0
111	13	Lac Ste Anne	F	M	33	101.5	2022	S		0
112	13	Lac Ste Anne	M	M	55	123.5	2022	S	0	0
113	13	Lac Ste Anne	M	M	45	115.5	2022	S	0	0
114	13	Lac Ste Anne	M	M	44	117.5	2022	S	0	0
115	4	Lac Ste Anne	M	M	92	146.5	2022	S	0	0
116	4	Lac Ste Anne	F	M	52	134	2022	S	0	0
117	4	Lac Ste Anne	F	M	60	121.5	2022	S	0	0
118	4	Lac Ste Anne	F	M	55	121	2022	S	0	0
119	1	Two Hills	M	M		123	2022	S		0
120	1	Woodlands	F	M	59	130	2022	I		0
121	6	Two Hills	M	M	50	115	2022	S	0	0
122	6	Two Hills	F	M	46	113	2022	S		0
123	6	Two Hills	M	M	43	120	2022	S	1	0
124	6	Two Hills	F	M	99	160	2022	S		0
125	6	Two Hills	M	M	72	135	2022	S	0	0
126	6	Two Hills	M	M	62	131	2022	S	0	0
127	1	Lac Ste Anne	M	M	80	136	2022	I	1	0
128	1	Lac Ste Anne	M	M	132	160	2022	I	0	0
129	6	aStrathcona	M	M	85	129	2022	S	0	0
130	6	aStrathcona	F	M	72	130	2022	S	0	0
131	6	aStrathcona	M	M	67	128	2022	S		0
132	6	aStrathcona	M	M	80	142	2022	S	0	0
133	6	aStrathcona	F	M	67	134	2022	S	0	0
134	6	aStrathcona	F	M	67	137	2022	S		0
135	8	Two Hills	F	M	47	103	2022	S	0	1
136	8	Two Hills	F	M	78	127	2022	S	0	0
137	8	Two Hills	F	M	63	127	2022	S	1	0

138	8	Two Hills	M	M	105	147	2022	S	0	0
139	8	Two Hills	F	M	111	150	2022	S	0	0
140	8	Two Hills	M	J	16	79.5	2022	S	0	0
141	8	Two Hills	F	J	17	81	2022	S	0	0
142	8	Two Hills	M	M	113	152	2022	S	0	0
143	34	Woodlands	F	M	85	143	2022	S	0	0
144	34	Woodlands	F	M	65	136	2022	S		0
145	34	Woodlands	F	M	58	130	2022	S	0	0
146	34	Woodlands	F	M	69	134.5	2022	S	0	0
147	34	Woodlands	F	M	58	130	2022	S	0	0
148	34	Woodlands	F	J	14	92	2022	S	0	0
149	34	Woodlands	F	J	6	55.5	2022	S	0	0
150	34	Woodlands	F	J	20	83	2022	S		0
151	34	Woodlands	F	J	17	84	2022	S	0	0
152	34	Woodlands	F	J	13	71.5	2022	S	0	0
153	34	Woodlands	F	J	17	78	2022	S	0	0
154	34	Woodlands	F	J	15	72	2022	S	0	0
155	34	Woodlands	F	J	15	75	2022	S	0	0
156	34	Woodlands	F	J	20	84	2022	S		0
157	34	Woodlands	F	J	16	78.5	2022	S	0	0
158	34	Woodlands	F	J	5.5	58	2022	S	0	0
159	34	Woodlands	F	J	12	69	2022	S	0	0
160	34	Woodlands	F	J	7	57.5	2022	S	0	0
161	34	Woodlands	F	J	18	79	2022	S	0	0
162	34	Woodlands	M	J	21	87	2022	S		0
163	34	Woodlands	M	J	17.5	90	2022	S	0	0
164	34	Woodlands	M	J	19.5	77	2022	S	0	0
165	34	Woodlands	M	J	15	73	2022	S		0
166	34	Woodlands	M	J	17	78	2022	S	0	0
167	34	Woodlands	M	J	19	82	2022	S		0
168	34	Woodlands	M	J	6	56	2022	S		0
169	34	Woodlands	M	J	6.5	58	2022	S	0	0
170	34	Woodlands	M	J	22	80	2022	S	0	0
171	34	Woodlands	M	J	18	79	2022	S	0	0
172	34	Woodlands	M	J	19	82.5	2022	S	0	0
173	34	Woodlands	M	J	18	75	2022	S	0	0
174	34	Woodlands	M	J	17.5	85.5	2022	S	0	0
175	34	Woodlands	M	J	19	86	2022	S	0	0
176	34	Woodlands	M	J	16	80	2022	S	0	0
177	4	Two Hills	M	M	57	127	2023	S	0	0
178	4	Two Hills	M	M	55	126	2023	S	0	0
179	4	Two Hills	M	M	63	118.5	2023	S	0	0
180	4	Two Hills	M	M	52	127.5	2023	S	1	0
181	4	Woodlands	F	M	38	111	2022	S	0	0
182	4	Woodlands	F	M	35	115	2022	S	0	0
183	4	Woodlands	M	M	45	117.5	2022	S	0	0
184	4	Woodlands	M	M	45	116.5	2022	S	0	0
185	4	Woodlands	F	M	46	121	2022	S	1	0
186	4	Woodlands	F	M	47	121.5	2022	S	1	0
187	4	Woodlands	M	M	57	120	2022	S	0	0
188	4	Woodlands	M	M	105	152.5	2022	S	0	0
189	2	Lac Ste Anne	F	M	90	143	2023	S	0	0
190	2	Lac Ste Anne	F	M	101	152.5	2023	S	0	0
191	3	Woodlands	M	M	47	113.5	2023	S		0
192	3	Woodlands	M	M	57.5	114	2023	S	0	0
193	3	Woodlands	F	M	51	112	2023	S	0	0
194	9	Woodlands	F	M	86.5	140	2023	S	0	0
195	9	Woodlands	M	M	27.5	94	2023	S	0	0
196	9	Woodlands	M	M	26.5	102	2023	S	1	0
197	9	Woodlands	M	M	27	106	2023	S	0	0
198	9	Woodlands	M	M	32	103	2023	S	0	0
199	9	Woodlands	F	M	34.5	103	2023	S	0	0
200	9	Woodlands	M	M	27	97.5	2023	S	0	0
201	9	Woodlands	M	M	24.5	98	2023	S	0	0
202	9	Woodlands	F	M	28	103	2023	S	0	0
203	2	Two Hills	F	J	17	83.5	2023	S	1	1
204	2	Two Hills	F	J	18	82.5	2023	S	1	1
205	1	aStrathcona	M	M	128	167	2023	I	1	0
206	4	Woodlands	M	M	60	120	2023	S	0	0

207	4	Woodlands	M	M	55	111	2023	S	0	0
208	4	Woodlands	M	M	55	118	2023	S	0	0
209	1	Woodlands	M	M	72	135	2023	I	1	0
210	1	Woodlands	M	M	74	131	2024	I	0	0
211	1	Woodlands	M	M	70	141	2024	I	0	0
212	1	Woodlands	M	M	94	151	2024	I	0	0
213	22	Lac Ste Anne	F	M	99	152	2024	S	0	0
214	22	Lac Ste Anne	F	M	67	132	2024	S	0	0
215	22	Lac Ste Anne	F	M	71	134	2024	S	0	0
216	22	Lac Ste Anne	F	M	74	114	2024	S	0	0
217	22	Lac Ste Anne	M	M	42	95	2024	S	0	0
218	3	Woodlands	M	M	62	122	2024	S		0
219	3	Woodlands	F	M	68	133	2024	S	0	0
220	3	Woodlands	M	M	63	135	2024	S		0
221	4	aStrathcona	M	M	63	131	2024	S	0	0
222	4	aStrathcona	M	M	65	139	2024	S	0	0
223	4	aStrathcona	M	M	76	145	2024	S	0	0
224	4	aStrathcona	M	M	67	147	2024	S	0	0
225	1	Woodlands	M	M	46	118	2024	I	0	0
226	2	Woodlands	F	M	62	124	2024	S	0	0
227	2	Woodlands	F	M	56	122	2024	S	0	0
228	1	Woodlands	M	M	73	132	2024	I	0	1
229	2	Woodlands	M	M	59	125	2024	S	0	0
230	2	Woodlands	F	M	59	132	2024	S	0	0
231	1	Woodlands	F	M	116	150	2024	I	1	1
232	3	Woodlands	M	M	73	125	2024	S	0	0
233	3	Woodlands	M	M	70	135	2024	S	0	0
234	7	Woodlands	M	M	66	127	2024	S	0	0
235	7	Woodlands	M	M	65	124	2024	S	0	0
236	7	Woodlands	F	M	60	121	2024	S	1	0
237	7	Woodlands	F	M	63	123	2024	S	0	0
238	7	Woodlands	F	M	63	131	2024	S	0	0
239	7	Woodlands	F	M	62	127	2024	S	0	0
240	7	Woodlands	F	M	48	124	2024	S	0	0
241	1	Woodlands	F	M	54	123	2024	I	0	0
242	16	Woodlands	M	M	25	97	2024	S	0	0
243	16	Woodlands	M	M	23	98	2024	S	0	0
244	16	Woodlands	M	M	22	92.5	2024	S	1	0
245	16	Woodlands	M	M	22	89	2024	S	0	0
246	16	Woodlands	F	M	21	101	2024	S	0	0
247	16	Woodlands	F	M	27	97	2024	S	0	0
248	16	Woodlands	M	M	23	97	2024	S	0	0
249	16	Woodlands	M	M	21	100	2024	S	0	0
250	16	Woodlands	F	M	22	90	2024	S	0	0
251	16	Woodlands	M	M	22	90	2024	S	0	0
252	16	Woodlands	F	M	28	100	2024	S	0	0
253	16	Woodlands	F	M	19	91.5	2024	S	0	0
254	16	Woodlands	M	M	32	96	2024	S	0	0
255	8	Woodlands	F	M	80	154	2024	S	1	0
256	8	Woodlands	F	M	50	123	2024	S	0	0
257	8	Woodlands	M	M	66	142	2024	S	0	0
258	8	Woodlands	M	J	3.5	50	2024	S	1	0
259	8	Woodlands	F	J	3.7	51	2024	S		0
260	8	Woodlands	M	J	3.92	51	2024	S	0	0
261	8	Woodlands	M	J	5.14	54	2024	S	1	0
262	3	Woodlands	F	M	40	114	2024	S		0
263	3	Woodlands	M	M	42	118	2024	S	0	1
264	3	Woodlands	F	M	40	113	2024	S	0	0
265	17	Woodlands	F	M	50	146	2024	S	0	0
266	17	Woodlands	F	M	54	135	2024	S	0	0
267	17	Woodlands	M	M	43	129	2024	S	0	0
268	17	Woodlands	F	M	36	116	2024	S	0	0
269	17	Woodlands	F	M	48	123	2024	S	0	0
270	17	Woodlands	F	M	34	100	2024	S	0	0
271	17	Woodlands	M	M	53	124	2024	S	0	0
272	17	Woodlands	F	M	26	101	2024	S	0	0
273	17	Woodlands	M	M	56	122	2024	S	0	0
274	17	Woodlands	F	M	48	127	2024	S	0	0
275	17	Woodlands	F	J	4.5	52	2024	S	0	0

276	17	Woodlands	M	J	6	55	2024	S		0
277	17	Woodlands	F	J	4.5	49	2024	S		0
278	17	Woodlands	F	J	5	51	2024	S	0	0
279	17	Woodlands	F	J	6.5	58	2024	S	0	0
280	17	Woodlands	M	J	4.5	52	2024	S		0
281	17	Woodlands	F	J	6	55	2024	S	0	0
282	8	Woodlands	F	M	76	137	2024	S		0
283	8	Woodlands	F	M	72	129	2024	S	0	0
284	8	Woodlands	F	M	94	140	2024	S	0	0
285	8	Woodlands	F	J	12	73	2024	S	0	0
286	8	Woodlands	F	J	10	67	2024	S	0	0
287	8	Woodlands	F	J	10	67	2024	S	0	0
288	8	Woodlands	F	J	8	61	2024	S	0	0
289	8	Woodlands	M	J	14	76	2024	S		0
290	1	Woodlands	F	M	50	124	2024	I	0	1
291	2	Woodlands	F	M	54	130	2024	S	0	0
292	2	Woodlands	F	J	8	65	2024	S		0
293	1	Woodlands	M	M	80	143	2024	I		0
